# The Reactivation of Immune Thrombocytopenic Purpura After SARS-CoV-2 Infection

**DOI:** 10.7759/cureus.44873

**Published:** 2023-09-07

**Authors:** Dheeraj Kumar Posa, Gilbert Roy Kamoga, Robert Walton

**Affiliations:** 1 Internal Medicine, White River Health System, Batesville, USA

**Keywords:** intravenous immunoglobulins (ivig), platelet count (plt), bleeding risk, sars-cov-2, viral itp

## Abstract

Immune thrombocytopenic purpura (ITP) is an autoimmune disease associated with bleeding symptoms and thrombocytopenia. It is diagnosed in patients with low platelet count after all the other causes of thrombocytopenia are ruled out. It can be presented as a primary condition, or it can be associated with other diseases. We report a case of ITP in a 65-year-old female with a one-day history of spontaneous bleeding gums, bruising, and petechiae all over her body. In further review of her history, it was noted that she had a history of ITP in remission and was recovering from a recent SARS-CoV-2 infection. We have excluded all the other causes of her thrombocytopenia, and we suspected that her viral illness would likely trigger this episode. Here, we report a case of ITP reactivation after SARS-CoV-2 infection.

## Introduction

A few cases of immune thrombocytopenic purpura (ITP) exacerbation following SARS-CoV-2 infection are reported in the literature. Chronic ITP is an immunological condition that leads to the destruction of platelets and impaired platelet production. Many triggers are associated with this condition, such as viral or bacterial infection, autoimmune diseases, and lymphoproliferative syndromes. The estimated prevalence of ITP in the United States is 9.5 per 100,000 people, with a global prevalence of over 200,000 people at any given time [1]. It shows bimodal age distribution with the highest prevalence in children aged <5 and also at the age of >60 because adults more frequently develop the chronic form of the disease. However, we present a case of a 65-year-old with profound thrombocytopenia (3,000/μL) following SARS-CoV-2 infection.

## Case presentation

**Figure 1:**
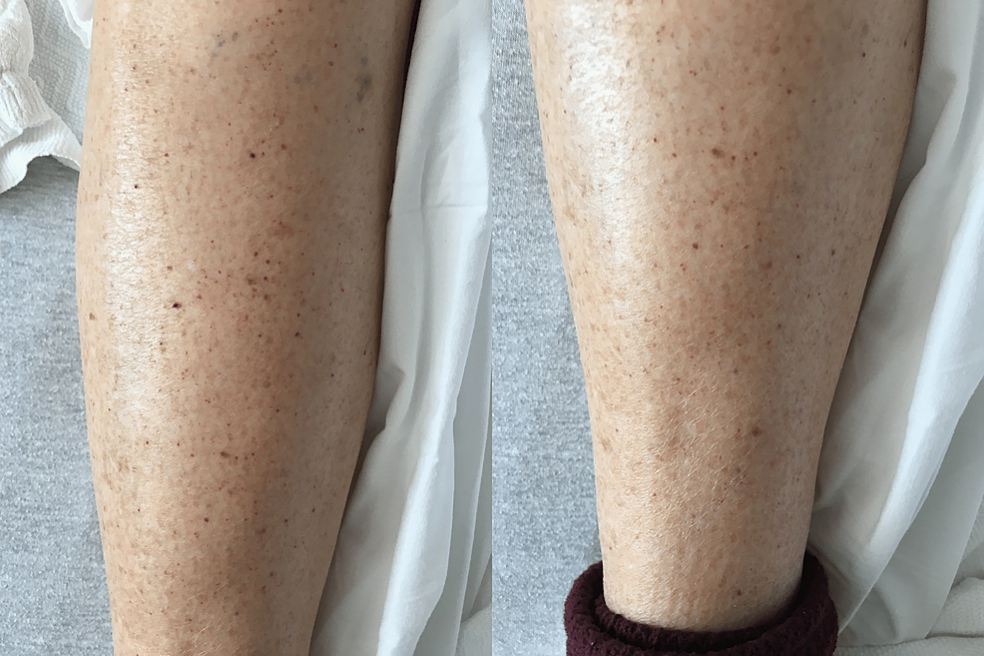
Multiple petechiae are seen on the bilateral lower extremities.

**Figure 2:**
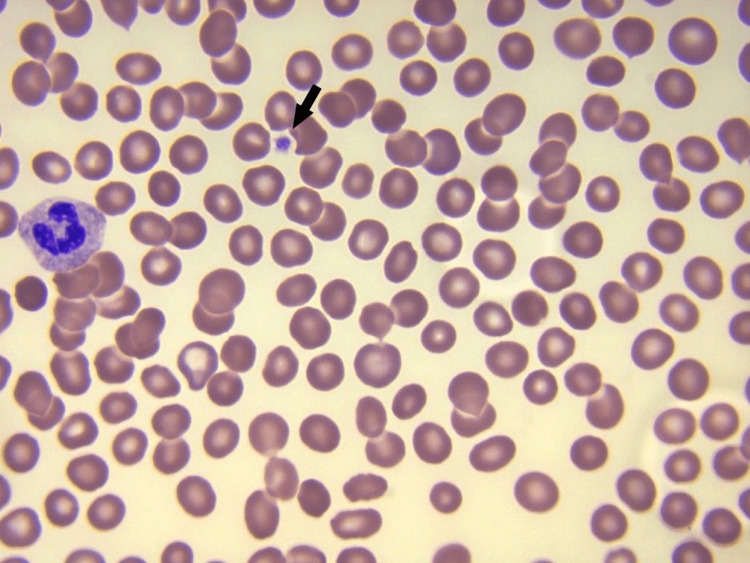
Peripheral blood smear showing single platelet with no megakaryocytes and normal RBC and WBC. RBC, red blood cell; WBC, white blood cell

A 65-year-old female with a history of immune thrombocytopenic purpura, hypothyroidism, and asthma presented with one day of spontaneous bleeding gums, bruising, and petechiae all over her body (Figure 1). On admission, her vital signs were stable, and laboratory findings revealed a white blood cell (WBC) of 4.8 K/μL (normal range: 4.8-10.8 K/μL), red blood cell (RBC) of 5.14 M/μL (normal range: 4.2-5.4 M/μL), hemoglobin of 14.5 g/dL (normal range: 12-16 g/dL), and platelet count of 3,000/μL (normal range: 130-400 K/μL). Coagulation studies were typical, with normal prothrombin time (PT), international normalized ratio (INR), and fibrinogen levels. Peripheral blood smear showed marked thrombocytopenia, no megakaryocytes, and normal white blood cell (WBC) and red blood cell (RBC) count and morphology (Figure 2).

She had a similar presentation when diagnosed with ITP four years ago, treated with intravenous immunoglobulin (IVIG) and steroids. However, she reported fatigue and sleeping more often than usual for the past few weeks. In addition, she recently had a SARS-CoV-2 infection a month ago, which made her quite ill and resolved without hospitalization. During this hospital visit, she denied any upper respiratory symptoms, and her oxygen saturation was >95% on ambient room air. Chest X-ray shows clear lung fields with no pneumothorax or pulmonary effusions. She denied any neurological issues, and her head CT showed no evidence of hemorrhage, mass, or edema. Her clinical presentation is consistent with the flare-up of chronic immune thrombocytopenic purpura secondary to a recent SARS-CoV-2 infection.

She received a unit of apheresed platelets and was treated with IVIG 0.5 g/kg/day for two days, followed by three days of IV methylprednisolone 1 g per day. She improved during the hospital course; by day 4, her platelet count had risen to 99 K/μL. She continued oral prednisone 1 mg/kg for a week, followed by a gradual taper completed in six weeks. She was discharged home with outpatient hematology follow-up.

## Discussion

The well-known mechanism that causes ITP is the production of autoantibodies against the platelet antigens (most likely immunoglobulin G {IgG} against glycoprotein {GP} IIb/IIIa and GP Ib/IX). These antibodies activate, complement, and cause phagocytosis by tissue macrophages located in the spleen and liver [2-4]. However, other proposed mechanisms were not well studied. SARS-CoV-2 is a viral infection that causes an excessive immune response and activates T-cell lines. There are a significant increase in the cytotoxic follicular helper cells and cytotoxic T helper (Th) cells responding to SARS-CoV-2 and a reduced proportion of reactive regulatory T-cells in these patients [5]. Studies have shown that ITP patients develop T-cell responses against platelet surface proteins, specifically on glycoprotein (GP) IIb/IIIa [6]. We assume that the molecular mimicry and cross-reactivity between the antibodies produced against the virus and the platelet antigens, followed by the activation of T-cells, could be the reason for the destruction of naïve platelets and the impaired production in the bone marrow.

The terminology for ITP is based on causation and inciting events. Primary ITP is an acquired autoimmune mechanism with no associated trigger, and secondary ITP is associated with a related trigger such as infection, autoimmune, and lymphoproliferative disorders [7]. In this case, we have excluded all the potential causes that lower the platelet level. So, we suspect that SARS-CoV-2 infection could trigger symptomatic thrombocytopenia in this patient. According to the American Society of Hematology (ASH) 2019 guidelines, the recommendations for hospital admission for patients with prior established diagnoses are highly variable [8]. Due to a profound drop of platelets to 3,000/μL and symptomatic bleeding, we expected that this patient would benefit from inpatient care and platelet transfusion. So, she was admitted and responded well by day 4 after augmented therapy with IVIG and systemic glucocorticoids.

The incidence of ITP increases with age and is more seen in females than males, with an average rate of 1.6-3.9 per 100,000 patient-years [9]. We suspect that the incidence of ITP does not depend on the severity of the infection but rather on a potential trigger that activates the immune response. We believe that the prevalence of ITP is higher in SARS-CoV-2 patients but less identified due to a relatively minor number of hospital admissions due to infection. We propose that patients who have tested positive for SARS-CoV-2, irrespective of the hospital admission, need a follow-up complete blood count by a primary care doctor within a month to check for platelet levels to avoid future fatal events.

## Conclusions

Severe thrombocytopenia leads to significant bleeding and may eventually lead to death. This case illustrates the condition's rarity and association with the recent pandemic. Hospital physicians and primary care physicians must create awareness among patients with an established diagnosis and newly diagnosed ITP. Early recognition and intervention could lead to prevention and better outcomes.
